# Investigation of Shear Behavior in High-Strength Bolt Connectors for Steel–Concrete Composite Beams

**DOI:** 10.3390/ma17246168

**Published:** 2024-12-17

**Authors:** Wei Li, Jie Wang, Xiaobo Xing, Huining Liu, Jin Di, Xianchao Sun, Leibo Li, Hongwei Li, Fengjiang Qin

**Affiliations:** 1 Shandong Expressway Qingdao Construction Management Co., Ltd., Qingdao 266300, China; liwei383768366@163.com (W.L.); xingxiaobo5507@163.com (X.X.); 2 Key Laboratory of New Technology for Construction of Cities in Mountain Area, School of Civil Engineering, Chongqing University, Chongqing 400045, China; wangjie205421@163.com (J.W.); dijin@cqu.edu.cn (J.D.); 3 Shandong Expressway Engineering Construction Group Co., Ltd., Jinan 250014, China; liuhuining6780@sina.com (H.L.); sunxianchao2640@sina.com (X.S.); lieibo1766@163.com (L.L.); lihongwei5607@sina.com (H.L.)

**Keywords:** high-strength bolt connector, shear behavior, interface friction, tension–shear behavior

## Abstract

High-strength bolt connectors, known for their robust strength and ease of disassembly, are suitable not only for the construction of new steel–concrete composite beams but also for reinforcing existing composite or steel beams. Static push-out tests were performed on nine specimens to examine their shear behavior. The primary failure mode was observed at the steel–concrete interface, characterized by the tensile–shear failure of the bolt and localized crushing of the concrete beneath the bolt. The preload had no significant influence on the ultimate bearing capacity and ultimate slip displacement, while it had a substantial impact on the initial slip load. The failure process was divided into static friction at the interface, sliding at the interface, elastic deformation of the bolt, and plastic deformation of the bolt. The parametric analysis using the finite element method was performed to assess the impact of concrete strength, reserved hole diameter, interface friction coefficient, and bolt diameter and strength. It revealed that the ultimate bearing capacity is composed of interfacial friction and bolt shear capacity, which are not independent of each other. To decouple these components, a novel calculation method for determining the ultimate bearing capacity of high-strength bolt connectors was developed and validated using existing test data.

## 1. Introduction

**Figure 1:**
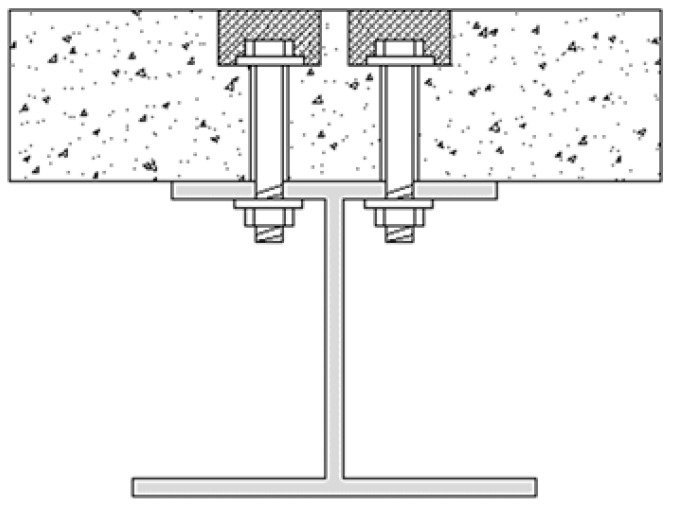
Diagram of high-strength bolt connector.

Steel–concrete composite beams leverage the advantages of both steel and concrete, offering high load-bearing capacity, enhanced stiffness, and excellent seismic performance [1]. These beams are widely used in long-span structures. The shear connector is crucial for ensuring the effective load transfer between the steel beam and concrete slab. Unlike traditional stud connectors, high-strength bolt connectors (see Figure 1) provide superior bonding strength, ease of installation, and the ability to be disassembled [2]. This makes them ideal for the efficient assembly of steel–concrete composite beams and facilitates convenient replacements during use. Additionally, the introduction of high-strength bolt connectors has significantly simplified the reinforcement process for existing steel–concrete composite beams and steel beams. These connectors are particularly advantageous in industrial buildings where rapid installation and maintenance are critical. High-strength bolt connectors have been widely employed in the seismic retrofitting of existing reinforced concrete buildings using external steel frames or steel exoskeletons [3,4,5,6,7,8]. Examples of such applications also include pedestrian overpasses, elevated roadways, prefabricated substations, and multi-story light steel structures [9].

Dallam [10] conducted tests on high-strength bolt connectors, revealing that these connectors exhibit a higher shear capacity compared to stud connectors of the same diameter. Dedic et al. [11] emphasized the suitability of high-strength bolt connectors for reinforcing steel–concrete composite beams with unknown steel strength, addressing the issue of the inadequate connection between steel beams and concrete slabs in existing structures. Kwon et al. developed a design approach for strengthening existing steel bridge girders by using post-installed shear connectors based on partial composite action [12] and found that high-strength bolt connectors outperform stud connectors in terms of fatigue performance, with a fatigue strength of approximately 240 MPa [13]. Chen et al. [14] investigated the impact of various factors on the shear performance of high-strength bolt connectors. Their study revealed that changes in bolt diameter significantly affect the ultimate bearing capacity. However, both the bolt diameter and preload have a minimal impact on the ultimate slip displacement. Specifically, for 16 mm diameter bolts, the preload has a negligible effect on the ultimate bearing capacity. In contrast, for 19 mm diameter bolts, the preload does influence the ultimate bearing capacity to some extent. The characteristics of the interface did not significantly affect either the ultimate bearing capacity or the slip displacement. Based on these findings, a mechanical model for high-strength bolt connectors was proposed.

Liu et al. [15,16,17] investigated the impacts of preload, bolt diameter and strength, steel flange hole spacing, and concrete strength on the ultimate bearing capacity and slip displacement of high-strength bolted connectors through numerical analysis. Zhao et al. [18] observed that high-strength bolt connectors typically fail due to concrete crushing and noted that reducing reserved hole diameters and increasing pad sizes can enhance their shear behavior. Zhang [19] investigated the shear performance and the calculated method for the high-strength bolts in steel–SFRC composite beams through test and numerical simulation. Xing et al. [9,20,21] demonstrated that high-strength bolt connectors enable steel–concrete composite beams to exhibit excellent recoverable performance. They also found that shot-peened steel and high-strength concrete are more suitable for detachable composite beams using high-strength bolt connectors. Based on test results, they derived calculation formulas for the friction coefficient at the interface, the shear bearing capacity of the bolt, and a practical formula for the applied load versus slip displacement curve for a single bolt connector.

Previous research has not yet developed a reliable formula for calculating the ultimate bearing capacity of high-strength bolt connectors, validated through both extensive experimental and simulation data. Additionally, there has been limited attention to the positive effects of interface friction and the negative effects of preload on the ultimate bearing capacity of bolts. Furthermore, current national codes [22,23,24,25] lack a standardized method for determining the ultimate bearing capacity of high-strength bolt connectors, which hinders their widespread adoption. The scarcity of existing studies further complicates the development of new standardized calculation methods.

To address these challenges, this paper conducts a comprehensive analysis and research effort to investigate the parameters influencing the shear behavior of high-strength bolt connectors, utilizing both experimental and finite element simulation methods. By considering the impact of interface friction and preload, a new calculation method for the ultimate bearing capacity is established based on the results of this study and previous research. This work not only provides valuable data but also offers theoretical support for the development of standardized design methods for high-strength bolt connectors.

## 2. Test Program

### 2.1. Specimen

**Figure 2:**
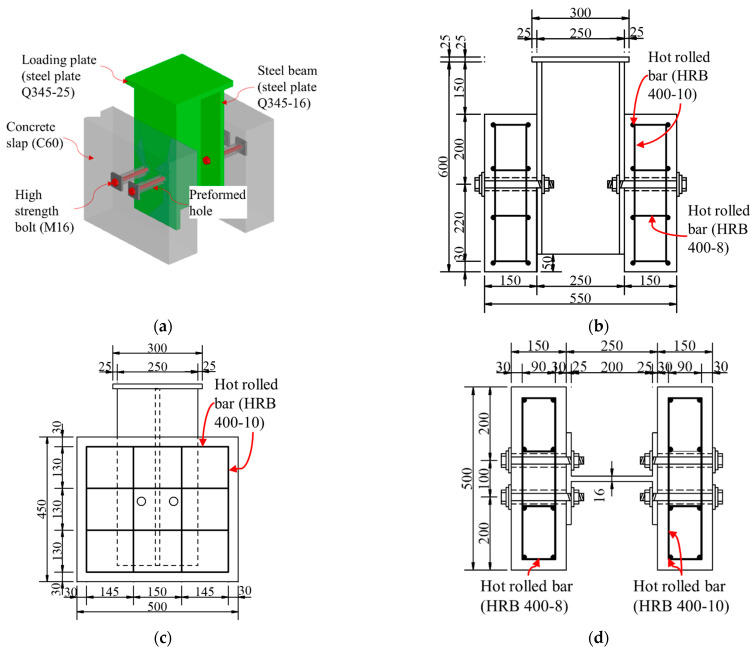
Dimension of specimens (unit: mm): (**a**) configuration; (**b**) front view; (**c**) side view; (**d**) vertical view.

To examine the influence of preload on the shear behavior of high-strength bolt connectors, three groups of push-out specimens were designed. Each group consisted of three identical specimens, with each group having a different level of bolt preload. The dimensions of all specimens were kept consistent, and the design followed the guidelines specified in EN 1994-1-1 [22], as illustrated in Figure 2. The main steel bars had a diameter of 10 mm, and the stirrups had a diameter of 8 mm. Circular holes were drilled in both the concrete slab and the steel beam. High-strength bolts, with a nominal ultimate tensile strength of 800 MPa and a nominal yield strength of 640 MPa, were inserted into these holes, and preload was applied to establish the connection between the steel beams and concrete slabs.

The torque values corresponding to different preloads were obtained according to GB50755-2012 [26], using a friction coefficient of 0.15 as specified by the bolt manufacturer. For M16 bolts, the calculated tightening torques for preload forces of 45 kN, 60 kN, and 75 kN are 108 N·m, 144 N·m, and 180 N·m, respectively. A torque wrench was utilized to tighten the bolts to these specified values. It is important to note that no material was used to fill the reserved holes, allowing for the easy replacement of high-strength bolts after failure. The parameters for each group of specimens are detailed in Table 1, with “*d_b_*” representing the diameter of high-strength bolts, “*d_c_*” and “ *d_s_*” representing the reserved holes diameters in concrete slabs and steel beams, respectively, and “*P*” denoting the preload. The specimen numbering convention is elucidated by the example “P45-1”, where “P45” indicates a preload of 45 kN, and “1” distinguishes between specimens with identical parameters.

According to practical engineering design, the diameter of the bolt holes in the steel beams was designed 2 mm larger than the bolt diameter. The processing accuracy for concrete is lower than that for steel beams, and concrete slabs are thicker. When there is an angular misalignment between the centerlines of the bolt holes in the concrete and those in the steel beam, smaller bolt hole diameters can lead to the bolts contacting the walls of the concrete holes after installation. To address this issue, the designed bolt-hole diameter of the concrete slab was made 2 mm larger than that of the steel beam. During construction, pre-embedded plastic pipes of the same diameter as the designed bolt holes were used to ensure that the dimensions of the concrete holes met the requirements. The steel beam served as part of the formwork for the concrete slab. Steel rods were used to connect the bolt holes in the steel beam with the pre-embedded plastic pipes, with the aim of aligning the bolt holes in the steel beam with those in the concrete slab as accurately as possible.

### 2.2. Material Properties

The material properties of the tested materials, including concrete (C60), steel plates (Q345-16 and Q345-25), hot rolled bars (HRB400-8 and HRB400-10), and high-strength bolts (M16), were determined according to GB/T 50081-2019 [27] and GB/T 228-2021 [28]. Specific values for material properties are detailed in Table 2.

For the concrete designated as “C60”, “C60” signifies a nominal prism compressive strength of 60 MPa. In the case of the steel plate labeled as “Q420-16”, “Q420” represents the nominal yield stress of 420 MPa, and “16” indicates a thickness of 16 mm. Regarding the hot rolled bar denoted as “HRB400-8”, “HRB400” signifies a nominal yield stress of 400 MPa, and “8” denotes a diameter of 8 mm. As for the high-strength bolt identified as “M16”, “M16” signifies a diameter of 16 mm. *f_cm_*, *f_cu_*, and *E_c_* represent the cubic compression strength, prism compressive strength, and elastic modulus of concrete, respectively. *f_vu_* and *E_v_* denote the shear strength and shear modulus of the high-strength bolt, respectively. *f_y_*, *f_u_*, and *E* denote the yield strength, tensile strength, and elastic modulus of steel, respectively. *ε_u_* denotes the strains corresponding to the tensile stress.

### 2.3. Test Setup and Measurement Arrangement

**Figure 3:**
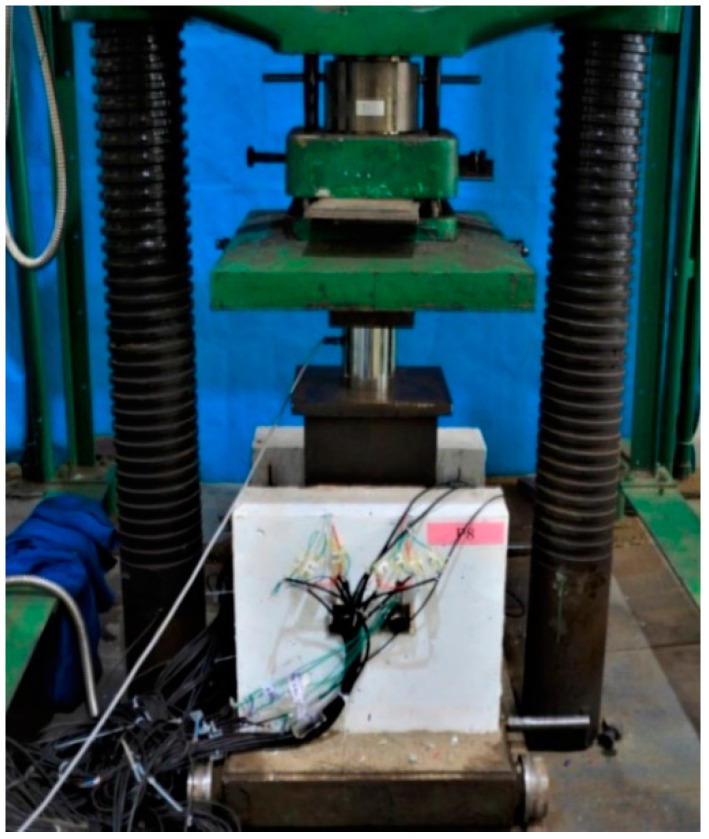
Test setup.

**Figure 4:**
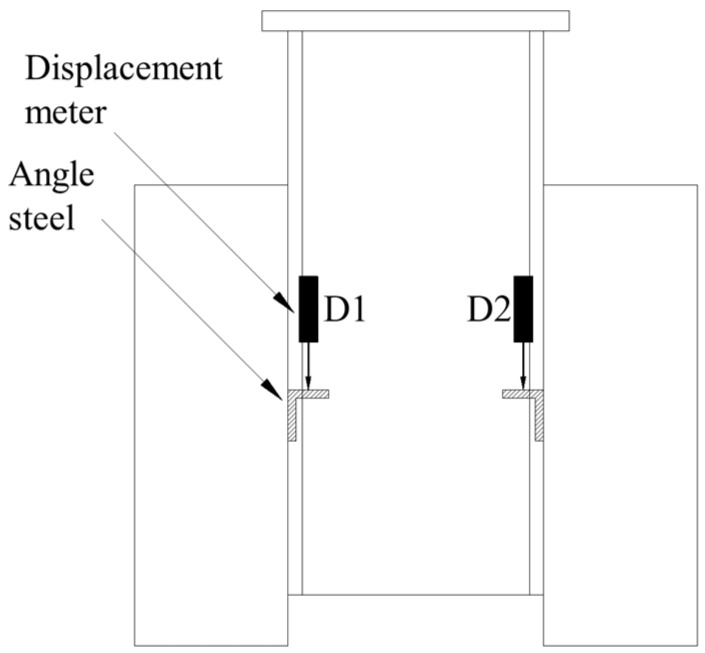
Layout of displacement meter.

A 5000 kN compression testing machine, fabricated by Tianshui Hong Shan Testing machine CO., LTD. (Tianshui, China), was used to perform the tests, as presented in Figure 3. A uniform pressure was applied on the loading plate of the specimen, and the bottom of the concrete slab was fixed on the floor. Four displacement meters were positioned at the same height as the high-strength bolt to monitor the relative slip between the steel beam and concrete slab. These meters were affixed to the steel beam. Angle steels were affixed to the concrete slab, and the measuring needle of the displacement meter was secured to the angle steel, as illustrated in Figure 4.

## 3. Test Results

### 3.1. Failure Mode

**Figure 5:**
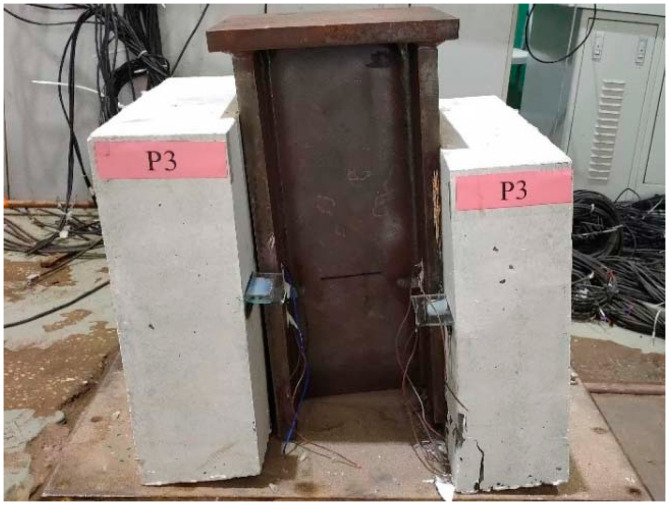
Failure mode.

Due to installation deviations of the specimen and variations in interface friction coefficients between its sides, slight differences in stress occur among the four high-strength bolted connectors within the same specimen. As a result, simultaneous cutting of all four high-strength bolts becomes unfeasible. Ultimately, all specimens experience single-sided bolt cutting as the final failure mode, as illustrated in Figure 5.

**Figure 6:**
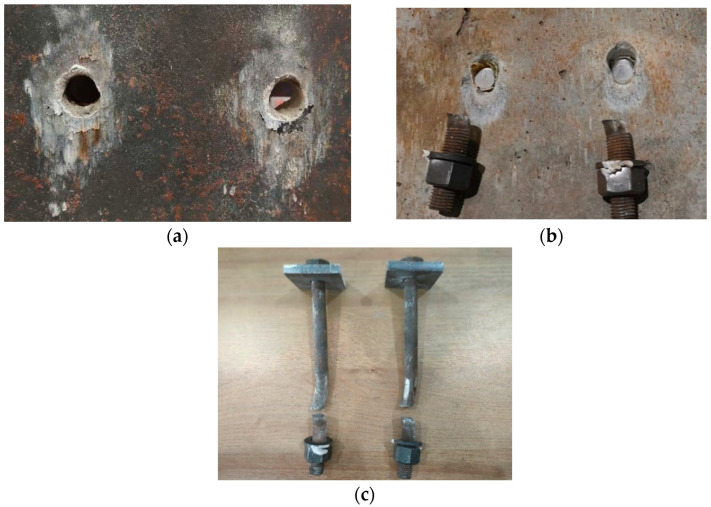
Failure mode: (**a**) steel beam; (**b**) concrete slab; (**c**) high-strength bolt.

The failure modes of the steel beams, concrete slabs, and high-strength bolts are illustrated in Figure 6. Compression occurred above the reserved hole in the steel beam during force transmission to the bolt, resulting in deformation. Partial crushing of the concrete below the bolt rod was observed due to the compressive effect. Additionally, the bending deformation of the bolt rod caused detachment from the concrete on its upper side. Significant shear failure was evident in the high-strength bolt, with bending deformation near the failure section and necking phenomena at the upper part. These observations indicate that the high-strength bolt primarily experiences shear force while also undergoing tensile force. The bending deformation resulted from localized crushing of the concrete beneath the bolt. Due to this bending deformation and preload, a notable tensile force developed at the upper part of the bolt section, leading to necking phenomena. However, no conspicuous deformations were observed in the remaining portions of the bolts.

### 3.2. The Load and Slip Displacement

**Figure 7:**
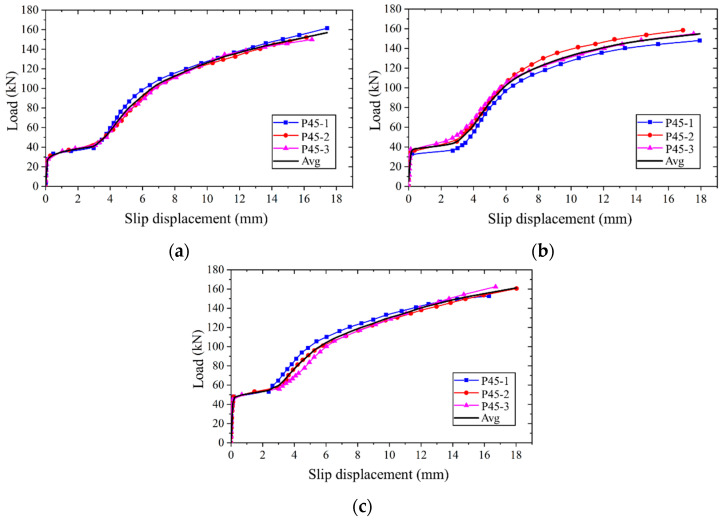
Applied load versus slip displacement curves: (**a**) specimen with preload of 45 kN; (**b**) specimen with preload of 60 kN; (**c**) specimen with preload of 75 kN.

The slip displacement is determined by averaging the readings from four displacement meters. The applied load versus slip displacement curves for the single bolt connector are illustrated in Figure 7. Due to the abrupt shear failure of high-strength bolts, the post-peak descending portion of these curves is unattainable. From these curves, key parameters such as the initial slip load (*P_f_*), ultimate bearing capacity (*P_u_*), and ultimate slip displacement (*δ_u_*) were identified. Table 3 summarizes the test results of single bolt connector and their mean values (*P_f,m_*, *P_u,m,_* and *δ_u,m_*).

**Figure 8:**
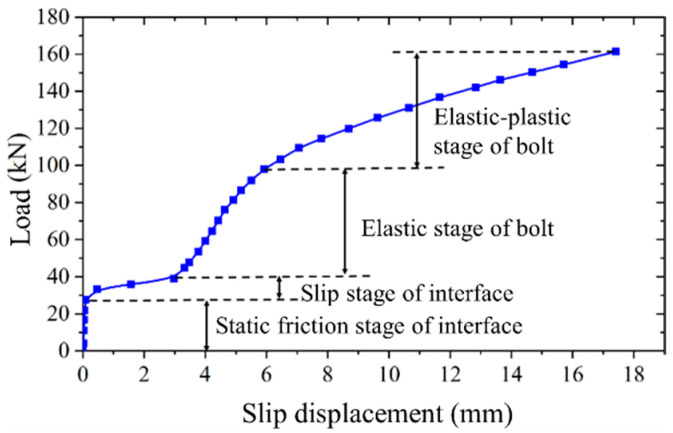
Loading behavior of high-strength bolt connector.

Analysis of Figure 7 and Table 3 reveals a notable consistency in the applied load versus slip displacement curves among the three identical specimens. A direct correlation is observed between the preload and the initial slip load; specifically, a 15 kN increase in preload results in an approximate 2.5 kN increase in the initial slip load. Interestingly, the preload does not influence the ultimate bearing capacity and ultimate slip displacement. The loading behavior of all specimens exhibits uniformity, characterized by four distinct stages, as delineated in Figure 8.

Static friction at the interface: When the applied load is relatively low, the primary load-bearing mechanism of the high-strength bolt connector is the static friction force at the interfaces. During this stage, the connector experiences minimal slip, which can be considered negligible. The static friction force acting at the interface is equivalent to the initial slip load.

Slip at the interface: When the applied load exceeds the static friction force at the interface, significant relative sliding occurs between the steel beams and the concrete slabs. This sliding is due to the gap between the reserved hole wall in the concrete slab and the bolt. The sliding continues until the bolt rod comes into contact with the reserved hole wall.

Elastic deformation of the bolt: When the bolt rod comes into contact with the reserved hole wall, the load is transmitted through the contact surface between the bolt and the reserved hole wall in the concrete slab. During this phase, the high-strength bolt experiences both tensile and shear forces, while the contact surface of the reserved hole in the concrete slab undergoes compression. The applied load versus slip displacement curves in this stage show an approximately linear relationship.

Plastic deformation of the bolt: With a continued increase in load, the bolt enters the plastic deformation phase, and the concrete beneath the bolt rod begins to crush progressively. During this phase, the stiffness of the high-strength bolt connector gradually diminishes until it ultimately fails through rupture. As the applied load versus slip displacement curve progresses in this stage, the slope gradually decreases, ultimately culminating in the high-strength bolt connector reaching its ultimate bearing capacity.

## 4. Numerical Analysis

### 4.1. Finite Element Model

**Figure 9:**
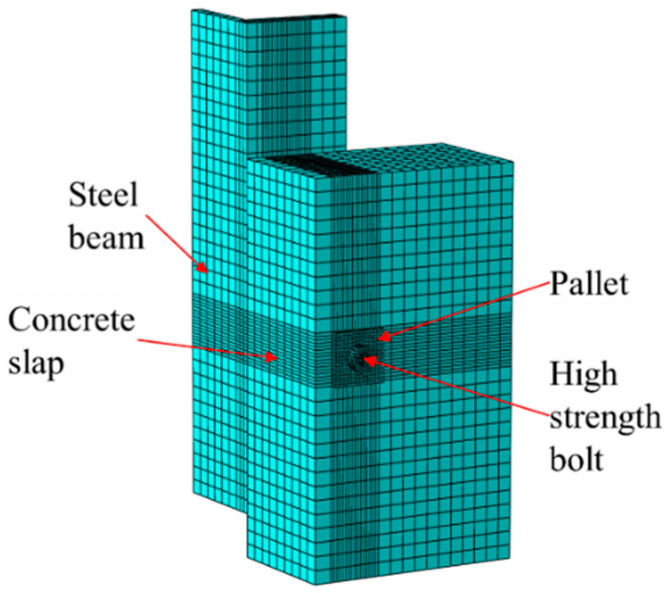
FEM.

The ABAQUS was used to establish a finite element model (FEM) of the specimen, as illustrated in Figure 9. The FEM consists of several components, including the steel beam, concrete slab, pallet, and high-strength bolt. To improve computational efficiency, a quarter-scale geometry model of the specimen was created, with symmetric boundary conditions applied to replicate the behavior of the entire specimen. The steel bars embedded within the concrete slab were modeled using truss elements (T3D2) and integrated into the concrete using the ‘Embedded Region’ command. The remaining components were simulated using solid elements (C3D8R). The element size for the high-strength bolt and its immediate surroundings was set to 3 mm, while the rest of the model used a 16 mm element size.

All interfaces between the various components were modeled as hard contacts, and the mechanical interactions at these interfaces were defined using hard contact and penalty function characteristics. The friction coefficient between the steel beams and the concrete slabs, set at 0.6, was calculated based on the initial slip load and preload obtained from tests. This value represents the average derived from the results of nine specimens. The preload was applied using the ‘Bolt load’ command, and a forced displacement was imposed on the top surface of the steel beam to simulate the applied load. To simulate the consolidation boundary of the specimen, the translational degrees of freedom for all nodes of the concrete slab bottom were constrained.

**Figure 10:**
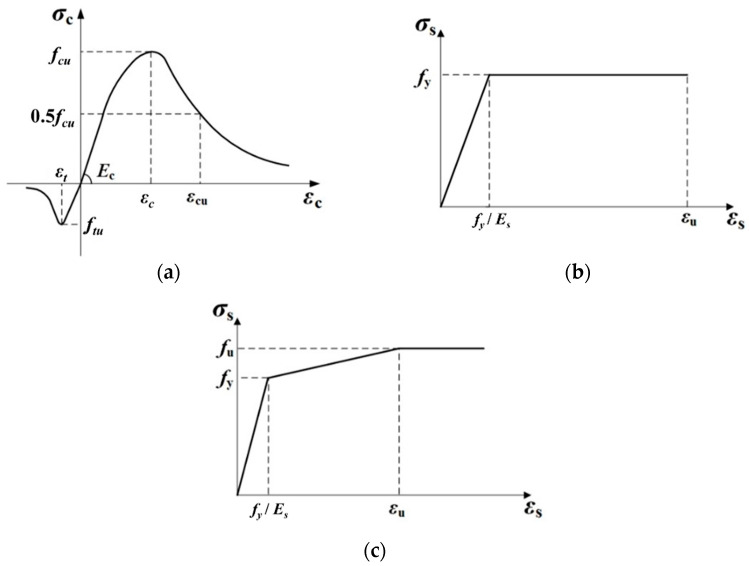
Material constitutive models: (**a**) concrete; (**b**) steel; (**c**) high-strength bolt.

The constitutive relationship for concrete in the FEM was determined in accordance with GB50010-2010 [29]. Uniaxial tensile and compressive damage coefficients were incorporated for concrete, featuring a gradual descent section to enhance convergence speed. The ideal elastic–plastic model was used as the constitutive behavior of steel without considering the strengthening effects. The multilinear kinematic hardening model was adopted in the high-strength bolt. In the FEM validation process, all material constitutive models were adjusted based on the measured data (refer to Table 2). The constitutive models of all used materials are shown in Figure 10.

### 4.2. FEM Verification

**Figure 11:**
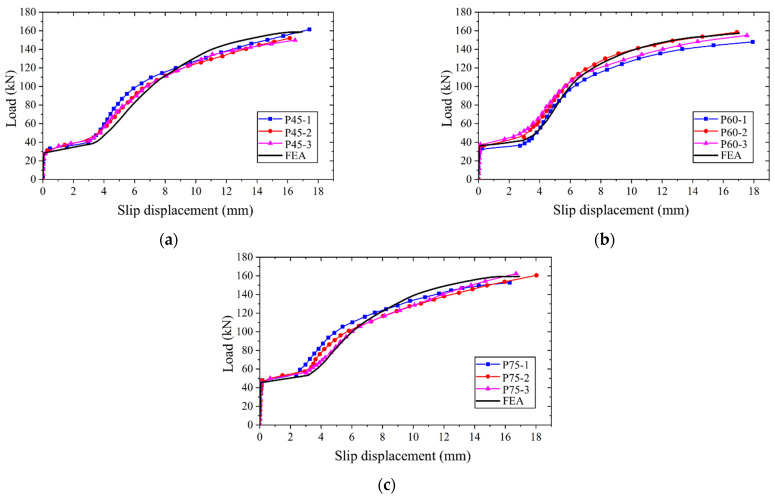
Comparison of simulated and test results: (**a**) specimen with preload of 45 kN; (**b**) specimen with preload of 60 kN; (**c**) specimen with preload of 75 kN.

The applied load versus slip displacement curve was calculated using the established FEM and subsequently compared with the experimentally measured curves, as depicted in Figure 11. It can be seen that the applied load versus slip displacement curve obtained through the FEM closely aligns with the test results. The disparity between the simulated and measured values of the ultimate bearing capacity falls within a 7% margin, while the difference in the ultimate slip displacement between the two sets of data is limited to 6%. These results underscore the capability of the established FEM in accurately replicating the loading behavior of high-strength bolt connectors, thus rendering it suitable for subsequent parametric analyses.

### 4.3. Analysis of the Failure Process of High-Strength Bolt Connector

**Figure 12:**
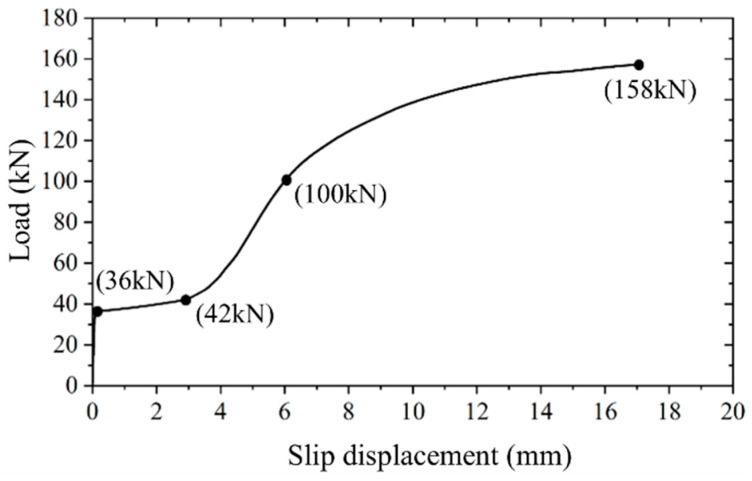
Applied load versus slip displacement curve of specimen P60-1.

**Figure 13:**
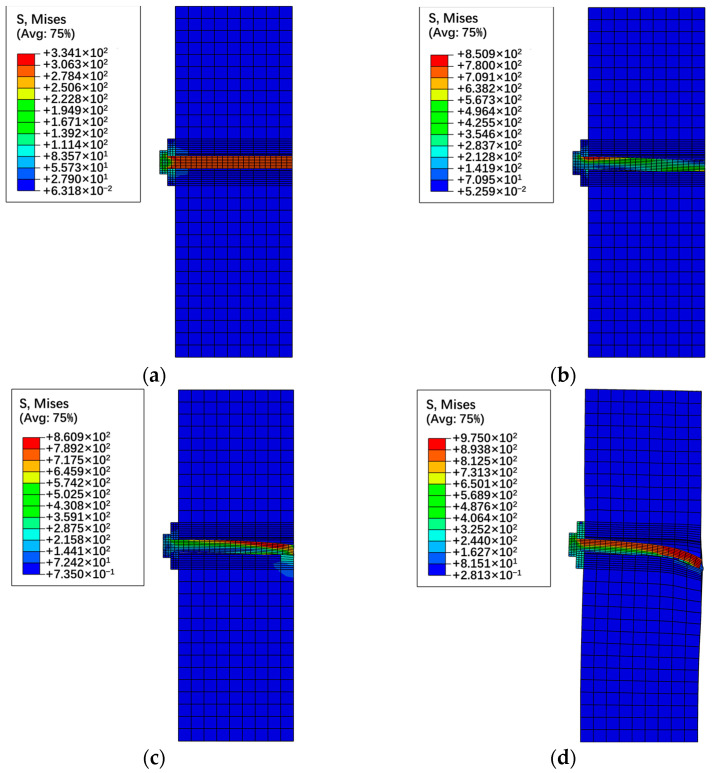
Failure behavior of high−strength bolt connector with applied load of (**a**) 36 kN; (**b**) 42 kN; (**c**) 100 kN; (**d**) 158 kN.

Section 3 offers a concise analysis of the failure process of high-strength bolt connectors, relying on experimental phenomena and results. However, due to the challenges in monitoring the stress and deformation of high-strength bolts during testing, conducting an in-depth analysis of their failure process proves difficult. Using specimen P60-1 as a case study, with the help of finite element simulation results, four typical loading points on its applied load versus slip displacement curve (refer to Figure 12) were selected to analyze the stress situation of high-strength bolts, as depicted in Figure 13.

It is obvious that, when the applied load reaches 36 kN, the failure process of the high-strength bolt connector transitions from the “Static Friction at the Interface” to the “Slip at the Interface”. At this point, the stress of the high-strength bolt is governed by the preload, exhibiting uniform tensile stress and minimal deformation. Subsequently, as the applied load reaches 42 kN, the bolt makes contact with the reserved hole wall. This causes the end of the bolt to be constrained by the concrete, resulting in a notable increase in tensile stress on the upper part of the bolt end, reaching the yield strength. Upon reaching an applied load of 100 kN, the failure process of the high-strength bolt connector shifts from the “Elastic deformation of the bolt” to the “Plastic deformation of the bolt”. The upper part of the bolt gradually yields and enters the plastic. At this juncture, the bolt exhibits cantilever bending deformation near the interface, with the upper surface stress near the interface reaching its maximum. As the applied load reaches 158 kN, the high-strength bolt connector reaches its ultimate bearing capacity and undergoes significant deformation. The stress throughout the section near the interface reaches the ultimate strength of 975 MPa, indicating failure of the bolt. Concurrently, due to excessive deformation of the connector, the concrete experiences severe compression, leading to significant deformation on the lower part of the reserved hole wall in the concrete slab.

### 4.4. Parametric Analysis

#### 4.4.1. Effect of Concrete Strength

**Figure 14:**
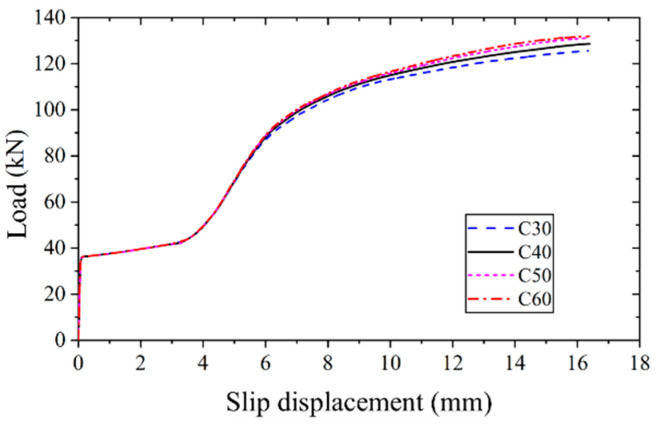
Applied load versus slip displacement curves of FEM of different concrete strengths.

The applied load versus slip displacement curves for high-strength bolt connectors with varying concrete prism compressive strength were computed using finite element analysis to investigate its influence on the shear behavior, as depicted in Figure 14. The results reveal that, as the concrete strength increases, the applied load versus slip displacement curves of high-strength bolt connectors exhibit a similar trend. The only notable difference observed is a slight increment in the ultimate bearing capacity, with the increase not exceeding 2 kN. Consequently, it can be inferred that the concrete prism compressive strength has a negligible impact on the shear behavior of the high-strength bolt connectors.

#### 4.4.2. Effect of Reserved Hole Diameter in Concrete Slab

**Figure 15:**
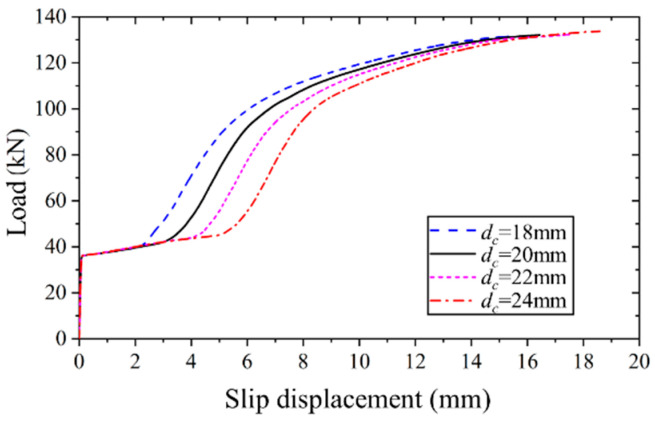
Applied load versus slip displacement curves of FEM with different reserved hole diameters.

The applied load versus slip displacement curves for high-strength bolt connectors with reserved hole diameters of 18 mm, 20 mm, 22 mm, and 24 mm were generated through finite element analysis, as illustrated in Figure 15. It is evident that an increase in the reserved hole size does not have any discernible impact on the initial slip load or ultimate bearing capacity. However, it does influence the slip displacement during the slip stage at the interface, consequently leading to a corresponding increase in the ultimate slip displacement. Specifically, for every 2 mm increase in the reserved hole diameter, the slip displacement experiences an approximately 1 mm increment.

#### 4.4.3. Effect of Friction Coefficient of Interface

**Figure 16:**
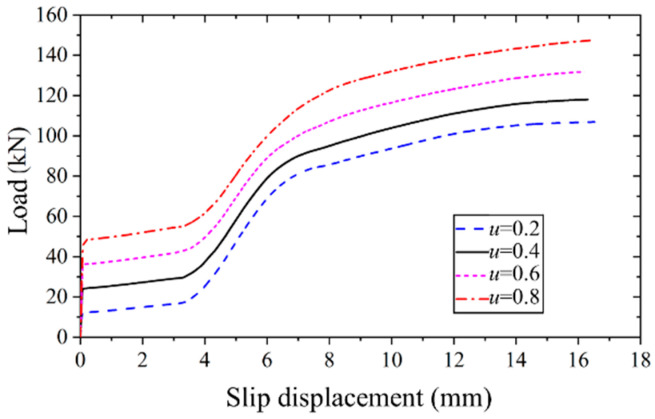
Applied load versus slip displacement curves of FEM with different friction coefficients.

Figure 16 illustrates the applied load versus slip displacement curves of high-strength bolt connectors with varying friction coefficients at the steel–concrete interface. It is evident that, as the friction coefficient increases, the initial slip load exhibits a linear increase, and the ultimate bearing capacity correspondingly rises. When the friction coefficient escalates from 0.2 to 0.8, the ultimate bearing capacity experiences a substantial 38.1% increase. Notably, the friction coefficient exerts no influence on the slip behavior.

Furthermore, it can be observed that the difference between the initial slip load and the ultimate bearing capacity remains essentially constant across different friction coefficients. This observation suggests that the ultimate bearing capacity of the high-strength bolt connector is a composite of the interfacial friction and shear capacity of the high-strength bolt, with these two factors exhibiting independence from each other.

#### 4.4.4. Effect of Bolt Diameter

**Figure 17:**
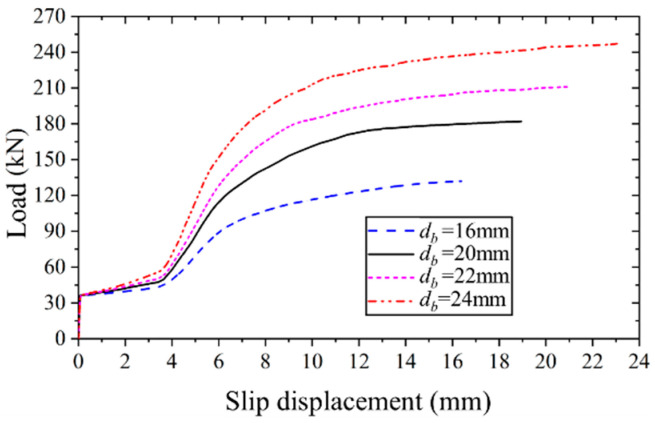
Applied load versus slip displacement curves of FEM with varying bolt diameters.

Figure 17 presents the applied load versus slip displacement curves for the high-strength bolt connector employing bolts of varying diameters. Notably, the ultimate bearing capacity and slip displacement of the high-strength bolt connector exhibit a nearly linear increase as the bolt diameter increases. Specifically, as the bolt diameter escalates from 16 mm to 24 mm, the ultimate bearing capacity and slip displacement experience substantial increments of 87.3% and 41.5%, respectively.

#### 4.4.5. Effect of Bolt Strength

**Figure 18:**
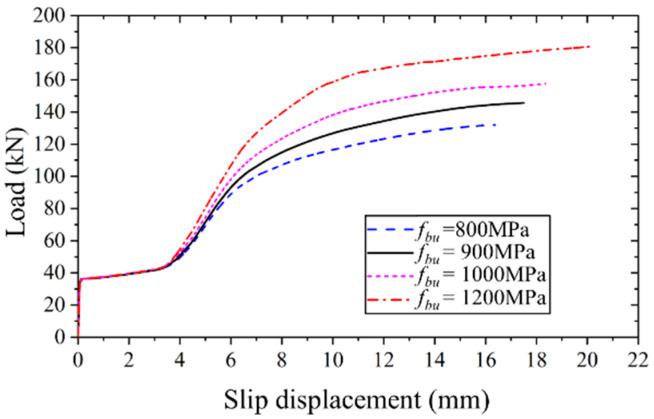
Applied load versus slip displacement curves of FEM with different bolt strengths.

Figure 18 depicts the applied load versus slip displacement curves for high-strength bolt connectors with varying tensile strengths, ranging from 800 MPa to 1200 MPa. It is evident that an increase in the bolt strength results in nearly linear growth in both the ultimate bearing capacity and slip displacement. Specifically, the ultimate bearing capacity and slip displacement increase by approximately 37.1% and 22.2%, respectively, as the bolt strength escalates from 800 MPa to 1200 MPa.

## 5. Calculation Method of Bearing Capacity of High-Strength Bolt Connector

### 5.1. Calculation Method

**Figure 19:**
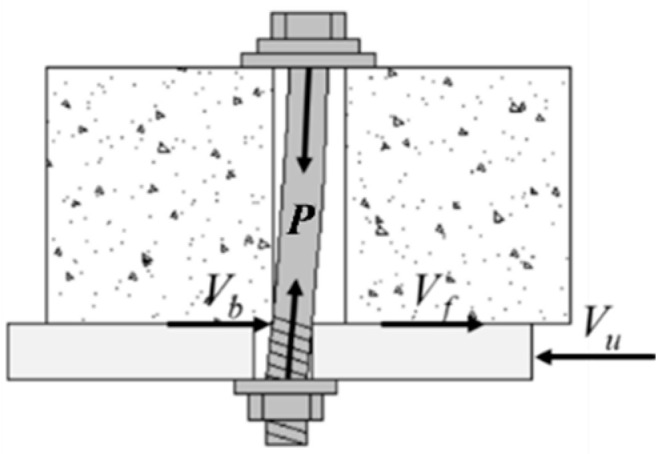
Mechanical model of high-strength bolt connector.

Based on the numerical analysis results, it is evident that the ultimate bearing capacity of the high-strength bolt connector comprises the interfacial friction and shear capacity of the high-strength bolt. The deformation and force experienced by high-strength bolt connectors under the influence of interfacial shear forces are illustrated in Figure 19, wherein the high-strength bolt operates in a tension–shear composite stress state. In accordance with GB50017-2017 [24], the bearing capacity of high-strength bolts subjected to tension–shear composite stress should satisfy the following formula:

(1) $$\sqrt{{\left(\frac{V_{b}}{\alpha_{1}A_{b}f_{u}}\right)}^{2}+{\left(\frac{P}{A_{b}f_{u}}\right)}^{2}}\leq 1.0$$

where *V_b_* denotes the shear capacity of the high-strength bolt; *α_1_* represents the correction factor of *V_b_*; *P* stands for the preload applied to the high-strength bolt; *A_b_* signifies the cross-sectional area of the high-strength bolt; and *f_u_* represents the tensile strength of the high-strength bolt.

Based on Formula (1), the shear capacity calculation formula for the high-strength bolt can be derived as follows:

(2) $$V_{b}=\alpha_{1}A_{b}f_{u}\sqrt{1-{\left(\frac{P}{A_{b}f_{u}}\right)}^{2}}$$

Based on Figure 19, the calculation formula for the ultimate bearing capacity of the high-strength bolt connector can be obtained as follows:

(3) $$V_{u}=V_{f}+V_{b}=\alpha_{1}A_{b}f_{u}\sqrt{1-{\left(\frac{P}{A_{b}f_{u}}\right)}^{2}}+\alpha_{2}\mu P$$

where *V_u_* represents the ultimate bearing capacity of the high-strength bolt connector; *V_f_* signifies the interface friction between the steel beam and concrete slab; *α*_2_ is the correction factor for interface friction.

**Figure 20:**
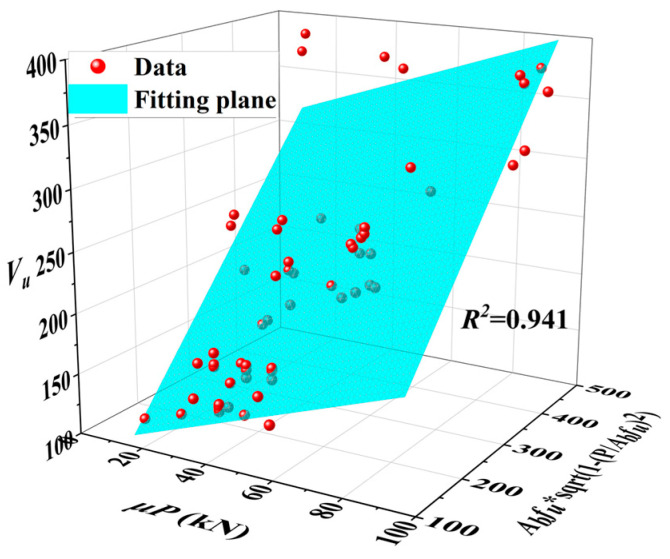
Fitting result.

Based on the test results of this paper, the results of parameter analysis, and the test results in Refs. [12,15,19,30,31], the least squares method was used to fit the correction factors, as depicted in Figure 20. The values of *α*_1_ and *α*_2_ are 0.64 and 1, respectively. The calculation formula for the ultimate bearing capacity of the high-strength bolt connector is as follows:

(4) $$V_{u}=\mu P+0.64A_{b}f_{u}\sqrt{1-{\left(\frac{P}{A_{b}f_{u}}\right)}^{2}}$$

### 5.2. Calculation Method Verification

Currently, there is no specific calculation method for determining the ultimate bearing capacity of high-strength bolt connectors outlined in the national codes. As a common practice, the calculation method typically employed for the ultimate bearing capacity of studs is adopted; this method is detailed in Table 4. Additionally, several alternative calculation methods proposed in the relevant pieces of literature for the ultimate bearing capacity of high-strength bolt connectors are also presented in Table 4.

**Figure 21:**
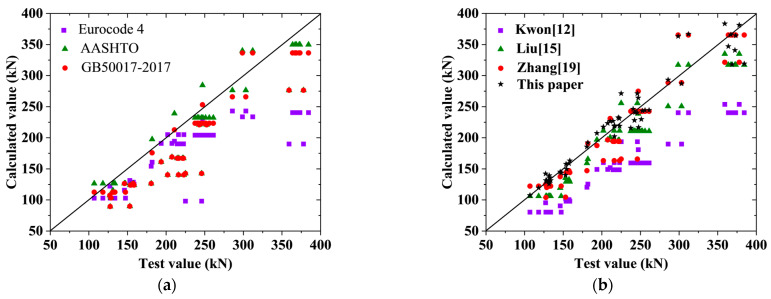
Comparison of the test results and calculated results: (**a**) codes; (**b**) references.

The ultimate bearing capacity of the high-strength bolt connectors in the tests and parameter analysis of this paper, as well as in previous research studies, is computed using the formulas provided in Table 4 and Formula (4). The resulting calculations are then compared with the corresponding test results, as depicted in Figure 21. Additionally, the average value (A.V.) and standard deviation (S.D.) of the ratios between the calculated and the test values were computed to evaluate the practicality of the calculation methods, as illustrated in Table 5.

It is evident that the Eurocode 4 method yields more secure estimates of ultimate bearing capacity, though it can be overly cautious for certain conditions. The AASHTO LRFD and GB50017-2017 methods generally provide secure estimates but exhibit significant variability, leading to potential safety concerns in some cases. The formula proposed by Kwon [12] tends to be overly conservative overall. Liu’s [15] method shows a higher degree of fit with experimental results and lower variability, although there are instances where it may not ensure safety. Zhang’s [19] method exhibits considerable variability relative to the test results, resulting in multiple unsafe scenarios.

Notably, Formula (4) proposed in this paper accounts for both interface friction and the tension–shear composite stress state of high-strength bolts. The results from Formula (4) demonstrate the best fit with the test values, exhibiting the smallest variability at just 6%. Although some unsafe conditions exist, the ideal calculation method for determining the ultimate bearing capacity of high-strength bolt connectors can be developed by applying an appropriate safety factor based on Formula (4), thereby accommodating safety redundancy requirements.

## 6. Conclusion

In this paper, the shear behavior of high-strength bolt connectors has been thoroughly investigated, leading to the following conclusions:

1. Under monotonic loading conditions, significant shear failure was evident in the high-strength bolt, with bending deformation near the failure section and necking phenomena at the upper part. The high-strength bolt primarily experiences shear force while also undergoing tensile force. Additionally, localized concrete beneath the bolt rod within the failure section was crushed, and compression deformation occurred above the reserved hole in the steel beam.
2. The loading process of high-strength bolt connectors can be delineated into four distinct stages: the static friction at the interface, followed by the slip at the interface, then the elastic deformation of the bolt, and, finally, the plastic deformation of the bolt.
3. When employing 8.8-grade high-strength bolts with a 16 mm diameter as connectors, the influence of concrete strength exceeding C30 on shear behavior can be negligible. The ultimate bearing capacity demonstrates an increase with the elevation of the interface friction coefficient and the bolt diameter and strength. The ultimate slip displacement escalates with the reserved hole diameter and the bolt diameter and strength. The initial slip load experiences an increase with the preload and interface friction coefficient.
4. Based on the findings from this paper and previous research studies, a calculation method for determining the ultimate bearing capacity of high-strength bolt connectors has been proposed. The calculated results align well with the test results, with variability at just 6%. When the calculation method designed for stud connectors is applied to compute the ultimate bearing capacity of high-strength bolt connectors, it is noted that the Eurocode 4 calculation method tends to be more secure, while The AASHTO LRFD and GB50017-2017 methods generally provide secure estimates but exhibit significant variability. Additionally, the formulas proposed by Kwon, Liu, and Zhang were also evaluated.

## Figures and Tables

**Table 1:** Parameters of specimens.

Specimen Number	*d_b_* (mm)	*P* (kN)	*d_c_* (mm)	*d_s_* (mm)
P45-1	16	45	20	18
P45-2	16	45	20	18
P45-3	16	45	20	18
P60-1	16	60	20	18
P60-2	16	60	20	18
P60-3	16	60	20	18
P75-1	16	75	20	18
P75-2	16	75	20	18
P75-3	16	75	20	18

**Table 2:** Material properties.

Material Number	*f_cm_* (MPa)	*f_cu_* (MPa)	*E_c_* (GPa)	*f_vu_* (MPa)	*E_v_* (GPa)	*f_y_* (MPa)	*f_u_* (MPa)	*E_s_* (GPa)	*ε_u_* (%)
C60	37.1	57.6	35.7	—	—	—	—	—	—
Q345-16	—	—	—	—	—	424.1	524.9	216.0	21.0
Q345-25	—	—	—	—	—	387.0	524.6	205.2	24.4
HRB400-8	—	—	—	—	—	441.0	644.5	203.1	21.7
HRB400-10	—	—	—	—	—	501.9	612.9	194.6	20.9
M16	—	—	—	685.0	77.7	851.0	975.0	205.1	16.3

**Table 3:** Test bearing capacity and sliding displacement of single bolted connection.

Specimen Number	*P_f_* (kN)	*P_u_* (kN)	*δ_u_* (mm)	*P_f,m_* (kN)	*P_u,m_* (kN)	*δ_u,m_* (mm)
P45-1	27.6	161.4	17.4	26.3	154.5	16.7
P45-2	23.4	152.1	16.2			
P45-3	27.8	150.0	16.5			
P60-1	36.2	148.0	17.9	36.8	153.7	17.4
P60-2	36.5	158.5	16.9			
P60-3	37.7	154.5	17.5			
P75-1	48.0	152.7	16.3	46.9	158.5	17.0
P75-2	47.9	160.5	18			
P75-3	44.8	162.4	16.7			

**Table 4:** Calculation method for ultimate bearing capacity in codes and references.

Code	Formula
Eurocode 4 [22]	$V_{u}=\min\left\{0.29\alpha d_{b}^{2}\sqrt{E_{c}f_{cu}}/1.25,\;0.64A_{b}f_{u}\right\}$
AASHTO LRFD [23]	$V_{u}=0.43A_{b}\sqrt{E_{c}f_{cu}}\leq 0.85A_{b}f_{u}$
GB50017-2017 [24]	$V_{u}=0.43A_{b}\sqrt{E_{c}f_{cu}}\leq 0.7A_{b}f_{u}$
Kwon [12]	$V_{u}=0.5A_{b}f_{u}$
Liu [15]	$V_{u}=0.66A_{b}f_{u}$
Zhang [19]	$V_{u}=\min\left\{0.5A_{b}\sqrt{E_{c}f_{cu}},\;0.76A_{b}f_{u}\right\}$

*α* is the ratio of the height to the diameter of the stud; when the ratio is greater than 4, *α* = 1.

**Table 5:** Calculation method for ultimate bearing capacity of stud connectors.

Code	Eurocode 4 [22]	AASHTO [23]	GB50017 [24]	Kwon [12]	Liu [15]	Zhang [19]	This Paper
A.V.	0.797	0.881	0.841	0.666	0.879	0.935	1.007
S.D.	0.120	0.137	0.110	0.060	0.079	0.102	0.070

## Data Availability

The original contributions presented in this study are included in the article. Further inquiries can be directed to the corresponding author(s).
